# Dynamic Heterogeneity of the Heart Valve Interstitial Cell Population in Mitral Valve Health and Disease

**DOI:** 10.3390/jcdd2030214

**Published:** 2015-08-17

**Authors:** Tori E. Horne, Matthew VandeKopple, Kimberly Sauls, Sara N. Koenig, Lindsey J. Anstine, Vidu Garg, Russell A. Norris, Joy Lincoln

**Affiliations:** 1 Center for Cardiovascular and Pulmonary Research and The Heart Center at Nationwide Children’s Hospital Research Institute, 575 Children’s Drive, Research Building III, WB4239, Columbus, OH 43215, USA; E-Mails: horne.119@buckeyemail.osu.edu (T.E.H.); matthew.vandekopple@nationwidechildrens.org (M.V.); Sara.Koenig@nationwidechildrens.org (S.N.K.); lindsey.miller@nationwidechildrens.org (L.J.A.); vidu.garg@nationwidechildrens.org (V.G.); 2 Department of Regenerative Medicine and Cell Biology, Medical University of South Carolina, Charleston, SC 29425, USA; E-Mails: saulsk@musc.edu (K.S.); norrisra@musc.edu (R.A.N.); 3 Department of Pediatrics, The Ohio State University, Columbus, OH 43215, USA

**Keywords:** mitral valve, valve interstitial cell, activation, Vimentin, Periostin, Twist1, smooth muscle α-actin

## Abstract

The heart valve interstitial cell (VIC) population is dynamic and thought to mediate lay down and maintenance of the tri-laminar extracellular matrix (ECM) structure within the developing and mature valve throughout life. Disturbances in the contribution and distribution of valve ECM components are detrimental to biomechanical function and associated with disease. This pathological process is associated with activation of resident VICs that in the absence of disease reside as quiescent cells. While these paradigms have been long standing, characterization of this abundant and ever-changing valve cell population is incomplete. Here we examine the expression pattern of Smooth muscle α-actin, Periostin, Twist1 and Vimentin in cultured VICs, heart valves from healthy embryonic, postnatal and adult mice, as well as mature valves from human patients and established mouse models of disease. We show that the VIC population is highly heterogeneous and phenotypes are dependent on age, species, location, and disease state. Furthermore, we identify phenotypic diversity across common models of mitral valve disease. These studies significantly contribute to characterizing the VIC population in health and disease and provide insights into the cellular dynamics that maintain valve structure in healthy adults and mediate pathologic remodeling in disease states.

## 1. Introduction

The mature heart valve leaflets must open and close over 100,000 times a day to maintain unidirectional blood flow through the heart. This is largely achieved by an intricate and highly organized connective tissue system within the valve structures that provide all the necessary biomechanical properties. The three stratified layers of extracellular matrix (ECM) within the valve leaflets (mitral, tricuspid) or cusps (aortic, pulmonic) are essential for stretch, compression and strength in response to changes in the hemodynamic load [1]. This ECM architecture is established during embryonic development and postnatal growth, and maintained by valve interstitial cells (VICs) that reside within the core of the valve leaflet in the adult. Overlying the ECM and VIC population is a layer of valve endothelial cells (VECs) that provide a physical barrier against the hemodynamic environment, and *in vitro* have been shown to molecularly communicate with underlying VICs to regulate their phenotype [2,3,4]. Maintaining this structure-function relationship of the valve leaflet is essential as changes in the contribution and distribution of ECM components lead to valve disease and associated biomechanical failure.

The VIC population is the most abundant cell type within the valve and predominantly originates from a subset of cells that undergo endothelial-to-mesenchymal transformation (EMT) in the atrioventricular canal and outflow tract regions. EMT results in formation of endocardial cushions that remodel and later give rise to the mature valve structures [5]. While endothelial-derived cells have been shown to be major source of valve precursor cells, there are several lines of evidence to suggest that additional sources also exist (reviewed [2]). *In vivo*, newly transformed mesenchyme cells express traditional markers including Twist1 and Snai1 and *in vitro*, these cells are immunoreactive to smooth muscle α-actin (SMA) (reviewed in [2]). Following EMT, the embryonic VICs (eVICs) continue to proliferate and mediate the remodeling process which involves breakdown of the primitive proteoglycans (e.g., hyaluronan) within the endocardial cushion and deposition of more specialized matrices (collagens, proteoglycans, elastin) that will give rise to the tri-laminar layers after birth. The specialized role of eVICs within the valve primordia suggests maturation beyond a typical endocardial cushion mesenchymal cell, however phenotypes of intermediate eVICs have not been explored. Soon after birth, the postnatal VICs are less proliferative and minimally active [6,7] and remain in this quiescent (qVIC) state in the absence of disease throughout life to maintain ECM homeostasis [1,6].

In contrast to healthy valves, there is a wealth of evidence to suggest that VICs are no longer quiescent in disease but activated (aVIC) similar to myofibroblasts. Studies in humans [6,8,9] large animals [7,10,11], and mice [12,13,14,15] have demonstrated that in disease states VICs upregulate the myofibroblast marker SMA, which is thought to recapitulate embryonic phenotypes. Notably, activation is not ubiquitous to all resident VICs suggesting heterogeneity amongst the population and there is a diverse profile of “activated” markers that are increased in subsets of VICs depending on disease, age, sex and species [16]. In myxomatous mitral valves from human patients, Rabkin *et al.* observed increased SMA in addition to Vimentin, Desmin and Embryonic Smooth muscle myosin heavy chain (SMemb) protein levels [6]. Similar increases in SMA and Desmin expression were also noted in myxomatous disease in a canine model [10]. However, differential changes in these markers were not observed at the mRNA level in a more recent non-biased screen of human myxomatous patients [17]. Histological analysis of human calcific aortic valve disease also report increased SMA in pediatric and adult patients [9], with an additional study by Latif *et al.*, describing aberrant Calponin, SM22, Caldesmon, and Smooth muscle myosin heavy chain expression, reflecting a more diverse VIC population containing myofibroblast- and smooth muscle-like lineages in this disease type [8]. In this later study, changes in smooth muscle and myofibroblast markers were regionalized within the tri-laminar layers of the ECM and changes in protein expression were not uniform to all aVICs, highlighting the complexity of differential VIC phenotypes in disease. While VIC activation is characteristic of several different forms of valve pathology, the stimulus and function of this process remain poorly understood.

The mechanisms that underlie VIC activation in disease are not fully understood, but there is increasing evidence from *in vitro* studies to suggest molecular, cellular and biomechanical influences. Culture of VICs on stiff substrates significantly increases SMA expression [18,19,20,21,22] more robustly after passage 1 [11] and Xu *et al.*, reported decreased SMA in confluent porcine VICs [23]. Similar to fibroblast activation, Tgfβ1 is the most potent inducer of SMA expression in cultured VICs [24,25,26], which is largely mediated by canonical Smad, and non-canonical MAPK signaling [27,28]. In addition, increased RhoA and Rho kinase activity is sufficient to promote myofibroblast-related phenotypic markers [29]. In contrast, FGF-2 [27], reduced β-catenin [30], and knockdown of the actin filament stabilizer, cofilin [11] have all been shown to prevent Tgfβ1- and substrate-induced VIC activation. Interestingly, substrate-induced VIC activation can be reversed by transferring cells to a soft substrate [18], or adding polyunsaturated fatty acids to the growth medium [31], highlighting the plasticity of cultured aVICs and potential reversibility of activation. At the cellular level, substrate-induced VIC activation is significantly attenuated when co-cultured with VECs both in the presence [32], or absence [33] of physical interactions. While the dogma has been that VIC activation marked by SMA expression is a key process in the onset of progression of heart valve pathology, the role of aVICs in disease has not directly been tested and therefore remains unknown.

It has been long presumed that aVICs function to promote ECM remodeling of the adult valve similar to the embryo, however, unlike development, ECM remodeling in disease is poorly controlled and often leads to biomechanical failure. SMA-positive aVICs have been shown to co-express matrix metalloproteinases (MMPs) that are thought to break down healthy ECM within the valve, and subsequently replace it with an alternative matrix dependent on the disease type [6]. For example, myxomatous mitral valves disease contains an abundance of proteoglycans and fragmented collagen fibers [34], which together contribute to prolapsed biomechanics. While in calcific aortic valve disease the matrix is mineralized [35] leading to leaflet stiffening and stenosis. In this latter disease state, VICs have been shown to express osteogenic markers, such as Runx2 and *in vitro* this “transdifferentiation” process precedes increased SMA [36,37]. Worthy of mention, SMA is not always increased in valve pathology and there are reports of unchanged or decreased expression [17,38,39], suggesting that VIC activation is not always present in disease, or SMA is not a ubiquitous marker of this “activated” phenotype. These collective studies highlight the complexity of VIC biology and while insights from *in vitro* studies are informative, little is known of the phenotypic characteristics of VICs *in situ* in health and disease.

In this study, we sought to build on several previous studies and determine the phenotypes of VICs in embryonic, postnatal, adult wild type and diseased heart valves by examining the expression patterns of Twist1, Vimentin, Periostin and SMA. In doing this, we highlight the complex heterogeneity of this abundant cell population in health and disease and emphasize the need to understand the role of VIC phenotypes in maintaining healthy valve structures and promoting disease pathogenesis. These studies provide important insights into the cellular mechanisms underlying disease that can be used in the development of alternative, non-surgical based therapies to prevent, attenuate, and potentially reverse pathological processes.

## 2. Experimental Section

### 2.1. Generation of Mice

*C57BL/6NJ* wild type mice were purchased from Jackson Labs (Bar Harbor, ME, USA) (stock number 005304) and embryos and hearts were collected at embryonic day (E) 12.5, 13.5, 17.5, postnatal days (PND) 2 and 5, 6 weeks and 12 months of age. Hearts from 12-month-old *Fbn1^C1039/+^* mice and *Fbn1^+/+^* (wild type littermate) mice were a kind gift from Vidu Garg and generated as previously described [40]. Tissue sections from 15 month *Tie2-cre*; *FilaminA^flox/flox^* (*Filamin A^−/−^*) and *Tie2-cre^−^*; *FilaminA^flox/flox^* (*Filamin A^+/+^*) mice were generously shared by Russell Norris [41].

### 2.2. Human Tissue

Human tissue was obtained from surgical specimens as part of a Leducq transatlantic network in collaboration with Russell Norris. All samples were harvested by offsite partners and were fixed, embedded and sectioned in their various laboratories. Sections were sent to The Research Institute at Nationwide Children’s Hospital for histological and immunohistological assessment. Consent and Institutional Review Board (IRB) approval for these studies is in place at partnering institutions. Control valve biopsies were obtained from patients who perished as a result of either subarachnoid hemorrhage or intracranial bleeds. No structural heart defects were noted in these patients. Myxomatous valves were surgical cases obtained from patients who fit diagnostic criteria of myxomatous degeneration and mitral valve prolapse. This was defined by >2 mm atrial leaflet displacement in a parasternal long-axis view as well as >5 mm valve thickness. Patient samples involved in the study were men greater than 50 years of age.

### 2.3. Histological Analysis

Human tissue, E12.5 and E13.5 whole embryos and hearts from E17.5, PND2, PND5, 6 weeks, 9 and 12 months old wild type, or diseased mice were fixed overnight in 4% paraformaldehyde at 4 °C and processed for paraffin embedding. Seven-micrometer-thick sections were cut, deparaffinized and subject to Pentachrome staining (American Master Tech Scientific, Inc.; Lodi, CA, USA) according to the manufacturer’s instructions. In sister sections, immunofluorescence was performed as previously described [42] by blocking tissue in 1% bovine serum albumin, 0.1% cold water fish skin gelatin, 0.1% Tween 20 and 0.05% NaN_3_/PBS for 1 h at room temperature. For double staining, anti-SMA (A2547, 1:200, Sigma-Aldrich, St. Louis, MO, USA) was incubated overnight at 4 °C with Periostin (Abcam ab14041, 1:100), Twist1 (Sigma T6451, 1:200) or Vimentin (ab45939, 1:200, Abcam, Cambridge, MA, USA). Non-specific binding was washed in 1× PBS and tissue sections were incubated in Donkey-anti mouse-568 (Molecular Probes-Life Technologies, Grand Island, NY, USA) for 1 h at room temperature to detect SMA expression, followed by Goat-anti rabbit-488 (Periostin, Twist1, Vimentin) under the same conditions. Tissue sections were then washed in 1× PBS and mounted with DAPI. For anti-Twist1, antigen retrieval was performed by boiling for 10 min in Unmasking Solution (Vector Labs, Youngstown, OH, USA) prior to blocking. Images were visualized using an Olympus BX51 and captured using CellSens software. Image brightness, contrast and removal of autofluorescent red blood cells were edited using Adobe Photoshop CS5.

### 2.4. Cell Culture

Two mitral valves (4 leaflets) were isolated from two 7-week old Sprague-Dawley rats (Taconic, Hudson, NY, USA) and briefly washed in 1× PBS. Leaflets were digested for 10 min in 5 mL type II collagenase solution (Worthington Biochemical, 600 U/mL in 10% FBS DMEM) to remove the endothelial cell layer. Leaflets were then placed in 10 mL collagenase overnight at 37 °C with gentle agitation. Homogenized cells were washed once with fresh VIC media (DMEM, 10% FBS, 1% pen/streptomycin) and all isolated cells were plated in a T-75 flask. Media was changed every 2–3 days, and cells were passaged when they reached ~80% confluence. Cells were used between passages 2–5.

One-hundred thousand rat mitral VICs (rMVICs) were plated on collagen-coated Matrigen Softwell (Matrigen, Brea, CA, USA) 6-well plates of varying stiffness (2 kPa, 12 kPa) or collagen-coated tissue culture plastic (TCP) and allowed to adhere for 24 h. Serum was then removed for 48 h, after which cells were briefly washed with 1× PBS and fixed in 4% paraformaldehyde. Fixed cell cultures were processed for immunostaining as described above.

## 3. Results

**Figure 1:**
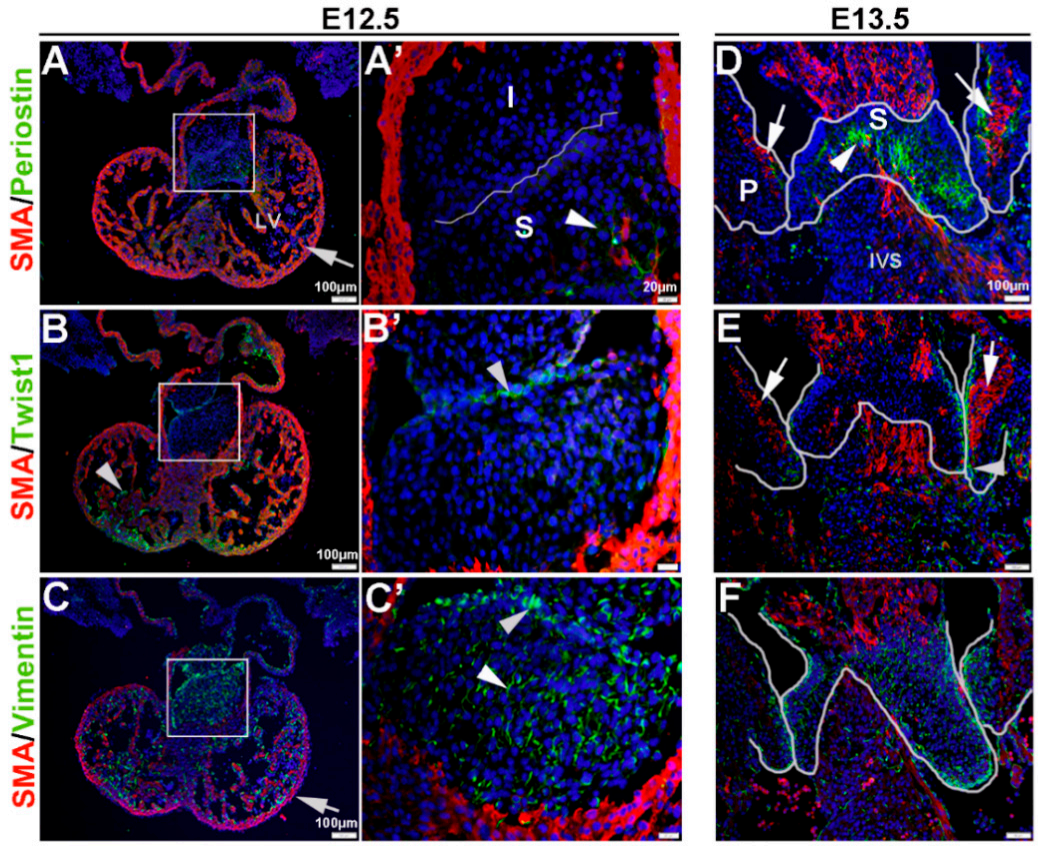
Characterization of VIC phenotypes during early heart valve development. Immunohistochemistry to show expression of smooth muscle α-actin (SMA) with Periostin (**A**,**A’**,**D**), Twist1 (**B**,**B’**,**E**) and Vimentin (**C**,**C’**,**F**) in cells within the inferior and superior endocardial cushions at E12.5 (**A**–**C’**) and atrioventricular valve primordia at E13.5 (**D**–**F**). Arrows indicate SMA expression (red) and arrowheads indicate Periostin (**A**,**A’**,**D**) Twist1 (**B**,**B’**,**E**) and Vimentin (**C**,**C’**,**F**) shown in green. The boxed areas in A-C are shown at higher magnification in (**A’**–**C’**). The white lines highlight the developing valve regions. I, inferior cushion; IVS, intraventricular septum; LV, left ventricle; P, parietal leaflets; S, superior cushion.

VICs have been shown to display differential molecular profiles *in vitro* and *in vivo* depending on age, sex and disease state [16,43]. The goal of this study was to examine VIC phenotypes based on expression of mesenchyme of Twist1, Vimentin, Periostin and SMA in healthy embryonic, postnatal and adult valves, as well as valves from established mouse models of valve disease. We show that mesenchyme cells within the endocardial cushion at E12.5 express very low levels of SMA (Figure 1A,A’), relative to the surrounding myocardium (arrow, Figure 1A), and Periostin is only detectable in the distal region of the superior cushion (arrowhead, Figure 1A,A’). Twist1 is observed in the endocardium within the ventricles (arrow, Figure 1B), and in the cushion protein expression is enriched in the endothelial cell layer although largely cytoplasmic (arrowhead, Figure 1B’). In contrast, Vimentin is highly expressed in the mesenchyme and endothelial cells within the cushion (arrowheads, Figure 1C,C’), as well as the endocardium (arrow, Figure 1C). At E13.5, SMA is observed within the core region of the parietal leaflets of the developing mitral and tricuspid valves (arrows, Figure 1D,E), and expression is distinct from Periostin localized towards the atrial and ventricular surfaces (Figure 1D). In the superior cushion, Periostin is more widespread throughout with a few cells co-localizing with SMA towards the center of the superior cushion (arrowhead, Figure 1D). At this time, Twist1 continues to be localized at the endothelium (arrowhead, Figure 1E), and Vimentin expression remains highly expressed throughout (Figure 1F).

**Figure 2:**
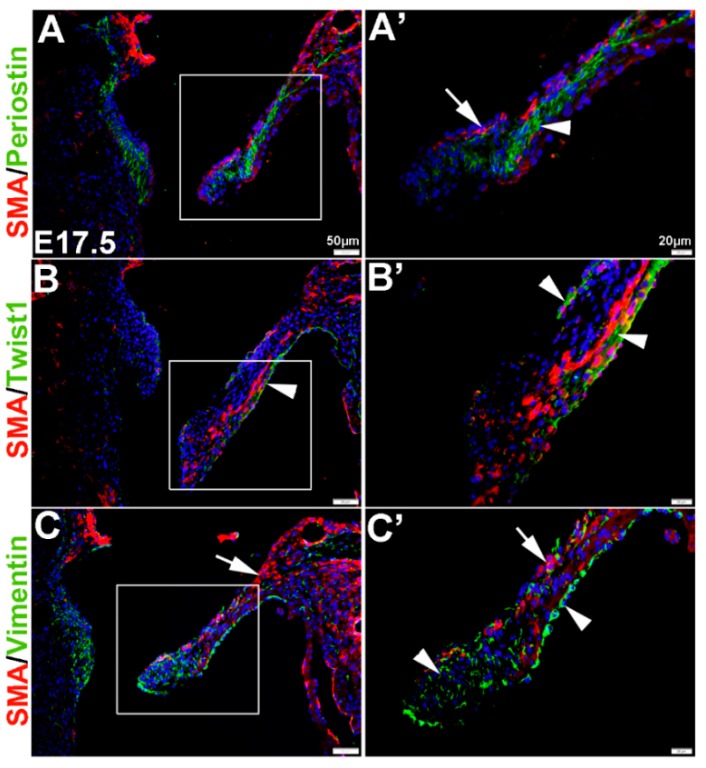
Characterization of VIC phenotypes in remodeling mitral heart valve structures. Immunohistochemistry to show expression of smooth muscle α-actin (SMA) with Periostin (**A**,**A’**), Twist1 (**B**,**B’**) and Vimentin (**C**,**C’**) in cells within the elongating mitral valve leaflet (mural). Arrows indicate SMA expression (red) and arrowheads indicate Periostin (**A**,**A’**) Twist1 (**B**,**B’**) and Vimentin (**C**,**C’**) shown in green. The boxed areas in A-C are shown at higher magnification in (**A’**–**C’**).

In the remodeling valve at E17.5, the eVICs have matured beyond a mesenchymal phenotype and are thought to be actively remodeling the ECM towards a tri-laminar structure. At this time, SMA is localized to the atrial (arrow, Figure 2A’) and ventricular (Figure 2B’) surface of the mural mitral valve leaflet, and noticeably SMA-positive cells are negative for Periostin seen throughout the leaflet core (arrowhead, Figure 2A’). Similar to E13.5, Twist1 remains enriched at the endothelium (arrowheads, Figure 2B’) and Vimentin is highly expressed in the majority of VECs and VICs (Figure 2C’).

These studies show that, *in vivo*, mesenchyme cells at E12.5 and eVICs of the remodeling valve widely express Vimentin, but are largely negative for SMA and Periostin until E13.5 when non-overlapping expression domains are observed. Twist1 however, is localized to the valve endothelium throughout valvulogenesis and low levels of expression are observed in the interstitial cell population.

**Figure 3:**
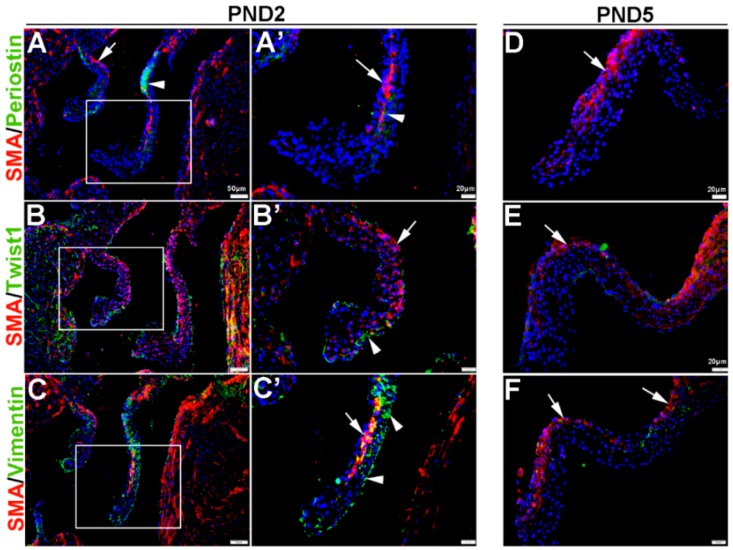
Characterization of VIC phenotypes in postnatal mitral valve leaflets. Immunohistochemistry to show expression of smooth muscle α-actin (SMA) with Periostin (**A**,**A’**,**D**), Twist1 (**B**,**B’**,**E**) and Vimentin (**C**,**C’**,**F**) in cells within the maturing mitral valve leaflet at postnatal day (PND) 2 (**A**–**C’**) and PND5 (**D**–**F**). Arrows indicate SMA expression (red) and arrowheads indicate Periostin (**A**,**A’**) Twist1 (**B**,**B’**) and Vimentin (**C**,**C’**) shown in green. The boxed areas in A–C are shown at higher magnification in (**A’**–**C’**).

**Figure 4:**
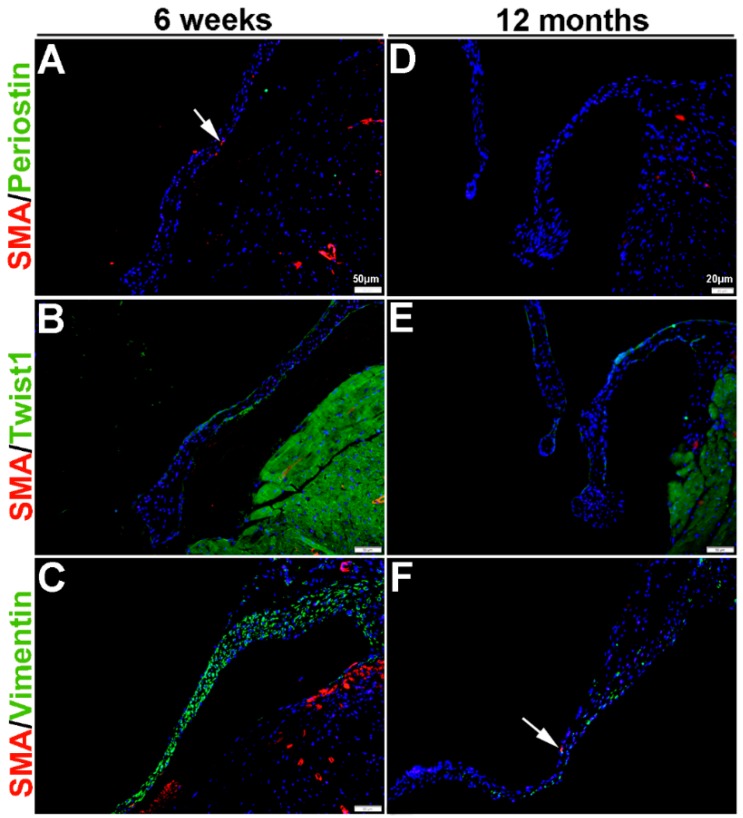
Characterization of VIC phenotypes in adult and aging mitral valves. Immunohistochemistry to show expression of smooth muscle α-actin (SMA) with Periostin (**A**,**D**), Twist1 (**B**,**E**) and Vimentin (**C**,**F**) in cells within the mature mitral valve leaflets. Arrows indicate SMA expression (red).

Once the tri-laminar structure has been established after birth, postnatal VICs become quiescent and remain this way throughout adulthood to maintain physiological valve structure and function [43]. Here we show that by PND2 and PND5, VICs continue to express SMA along the atrial surface of the mitral valve leaflets (arrows, Figure 3A–F). While Periostin is downregulated by PND5, as is Twist1 although low levels are still detected in the endothelium (arrowheads, Figure 3B’,E). Vimentin remains highly detectable in most, but not all VICs and VECs at PND2 and many positive VICs co-express SMA (Figure 3C,C’). By PND5, Vimentin is notably downregulated (Figure 3F). At 6 weeks and 12 months of age, Periostin is undetected (Figure 4A,D) and SMA expression is rare (Figure 4A–F). Twist1 remains endothelial at both these later time points (Figure 4B,E), and Vimentin appears higher at 6 weeks compared to PND5, however by 12 months immunoreactivity is observed in few cells (Figure 4F). In conclusion, Vimentin commonly marks the VIC and VEC population at all stages prior to 12 months, while Twist1 is largely cytoplasmic and enriched at the endothelium. SMA and Perisotin are detectable from E13.5 and mark distinct and localized VIC populations until postnatal growth and remodeling are complete.

**Figure 5:**
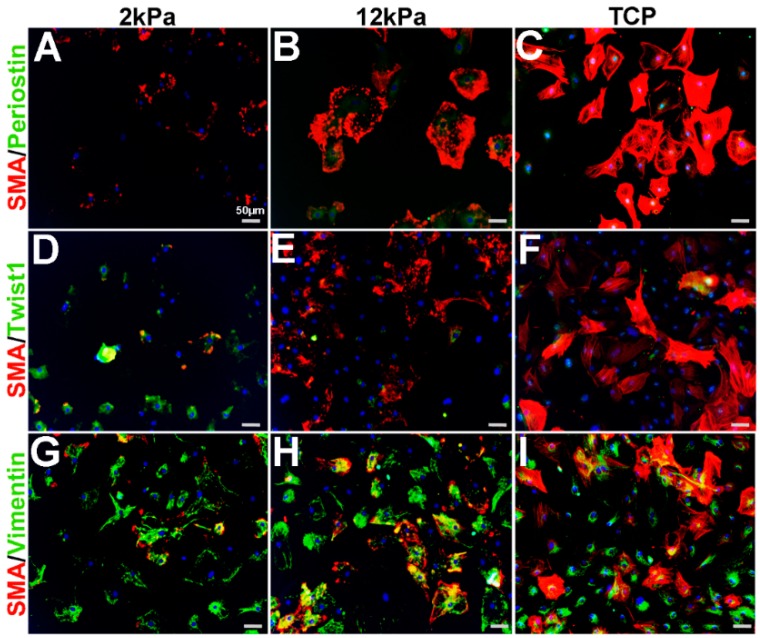
Expression profiles of rat mitral VICs cultured on increasing substrate stiffness. Immunohistochemistry to show expression of smooth muscle α-actin (SMA) with Periostin (**A**–**C**), Twist1 (**D**–**F**)) and Vimentin (**G**–**I**) in isolated rat mitral VICs cultured on 3D nanofiber (polycaprolactone) scaffolds coated with collagen (Nanofiber Solutions) with substrate rigidities of 2 kPa (**A**,**D**,**G**), 12 kPa (**B**,**E**,**H**) and tissue culture plastic (TCP) (**C**,**F**,**I**).

Similar to cardiac fibroblasts, VICs spontaneously express SMA when cultured on stiff substrates such as tissue culture plastic (TCP) and considered activated [11,18,19,20,21,22]. Furthermore, cell passage number and density can further affect SMA levels [11,23]. To characterize the phenotype of cultured VICs relative to observations made from *in vivo* studies (Figure 1, Figure 2, Figure 3 and Figure 4), adult rat mitral VICs (rMVICs) were isolated and 100,000 cells were cultured at passage number 2–5 on 3D nanofiber (polycaprolactone) scaffolds coated with collagen (Nanofiber Solutions) with substrate rigidities of 2 and 12 kPa, in addition to collagen-coated tissue culture plastic (TCP). As shown in Figure 5, rVICs cultured on 2 kPa express low levels of SMA and Periostin (Figure 5A), while nuclear Twist1 is prominent (Figure 5D) and Vimentin is highly expressed (Figure 5G). Similar to *in vivo* studies (Figure 1, Figure 2, Figure 3 and Figure 4), Vimentin continues to be expressed in VICs under all conditions and overlaps with SMA expression that increases and forms stress fibers on stiffer substrate surfaces (12 kPa, TCP) (Figure 5G–I). In contrast, Periostin remains at low levels around the nuclei on 12 kPa and TCP substrates (Figure 5B,C) and Twist1 expression is reduced with substrate stiffness (Figure 5E,F). These data suggest that on softer substrates, rMVICs are Twist1 and Vimentin positive and morphologically undifferentiated, while as stiffness increases, VICs increase SMA and form organized actin stress fibers.

**Figure 6:**
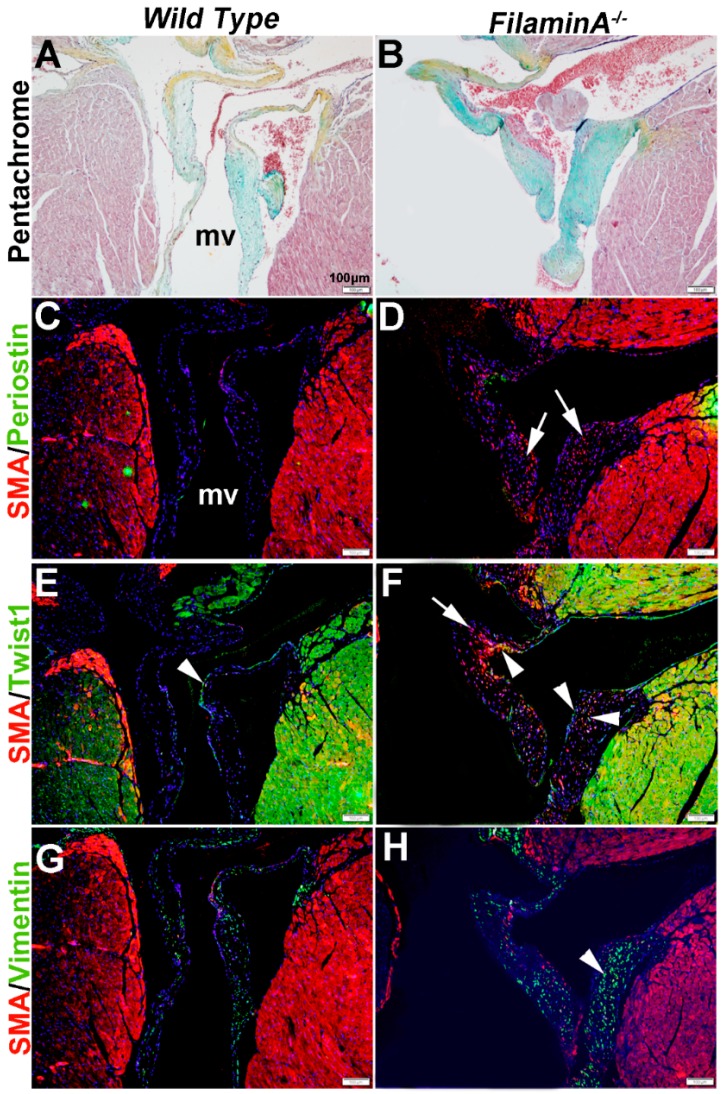
Characterization of VIC phenotypes in myxomatous mitral valves from 15 month old *Filamin A^−/−^* mice. Pentachrome staining to show extracellular matrix composition and organization in 15 month wild type (*Filamin A*^+*/+*^) (**A**) and *Filamin A^−/−^* (**B**) mice. (**C**–**H**) Immunohistochemistry to detect expression of smooth muscle α-actin (SMA) with Periostin (**C**,**D**), Twist1 (**E**,**F**) and Vimentin (**G**,**H**) in cells within the mitral valve leaflet of wild type (**C**,**E**,**G**) and *Filamin A^−/−^* (**D**,**F**,**H**) mice. Arrows indicate SMA expression (red) and arrowheads indicate Twist1 (**E**,**F**) and Vimentin (**G**,**H**) shown in green. mv: mitral valve

In heart valve disease, changes in VIC phenotypes have been described [6,43]. Here, we examine VIC phenotypes in established mouse models of mitral valve disease using SMA, Periostin, Twist1 and Vimentin expression. *Filamin A^−/−^* mice develop myxomatous mitral valve disease by 2 months of age, characterized by significant leaflet thickening and pathologic ECM remodeling (Figure 6A,B) [41].

At 15 months of age, these myxomatous changes are associated with increased proteoglycans (Figure 6B). In addition, SMA is detected in subsets of VICs but not all (arrows, Figure 6D), and increased Twist1 appears to be ectopically expressed in SMA-positive VICs (arrowheads, Figure 6F). Periostin was not significantly detected in wild type or diseased mice (Figure 6C,D) and Vimentin expression appears similar (Figure 6G,H).

**Figure 7:**
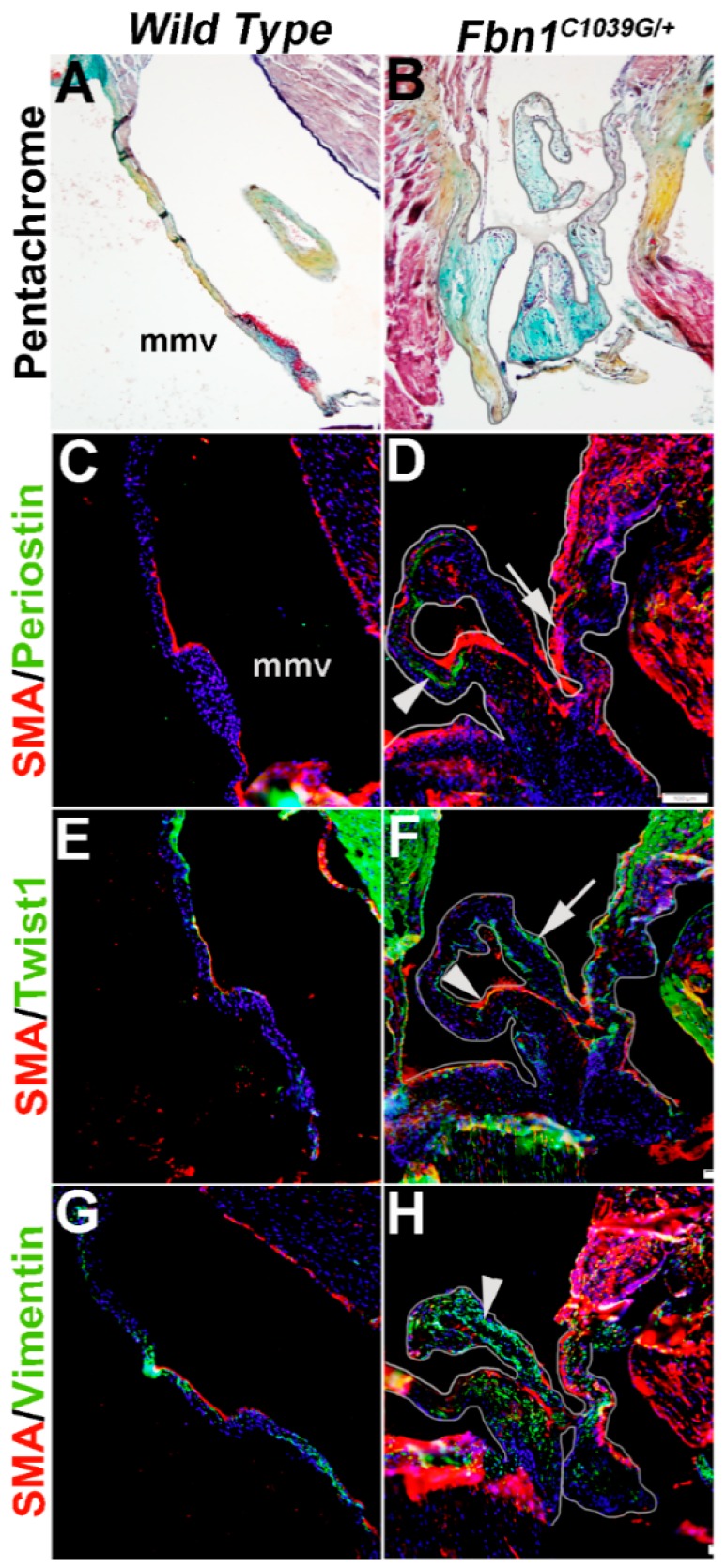
Characterization of VIC phenotypes in diseased mitral valves from 12 month old *Fbn1^C1039/+^* mice. Pentachrome staining to show extracellular matrix composition and organization in 12 month wild type (*Fbn1^+/+^* mice) (**A**) and *Fbn1^C1039/+^* (**B**) mice. (**C**–**H**) Immunohistochemistry to detect expression of smooth muscle α-actin (SMA) with Periostin (**C**,**D**), Twist1 (**E**,**F**) and Vimentin (**G**,**H**) in cells within the mitral valve leaflet of wild type (**C**,**E**,**G**) and *Fbn1^C1039/+^* (**D**,**F**,**H**) mice. Arrows indicate SMA expression (red) and arrowheads indicate Periostin (**C**,**D**), Twist1 (**E**,**F**) and Vimentin (**G**,**H**) shown in green. mmv, mural mitral valve.

Mice carrying a cysteine substitution mutation in *Fibrillin-1* (*Fbn1*) serve as a model for Marfan Syndrome. Homozygous *Fbn1^C1039/C1039^* mice die soon after birth from aortic dissection, but heterozygotes are viable and exhibit mitral valve thickening and functional prolapse by 9 months of age [40]. In Figure 7, we show that in diseased mitral valves from *Fbn1^C1039/+^* mice SMA (arrow, Figure 7D) and Periostin (arrowhead, Figure 7D) are increased in different VIC subsets. In addition, Twist1 reactivity is also increased at the endothelium (arrowhead, Figure 7F). Vimentin remains highly expressed in the majority of VICs in *Fbn1^C1039/+^* mice (arrowhead, Figure 7H), overlapping with SMA expression in several places. These observations made from Figure 6 and Figure 7 highlight the diversity of VIC phenotypes in mouse models of mitral valve disease.

**Figure 8:**
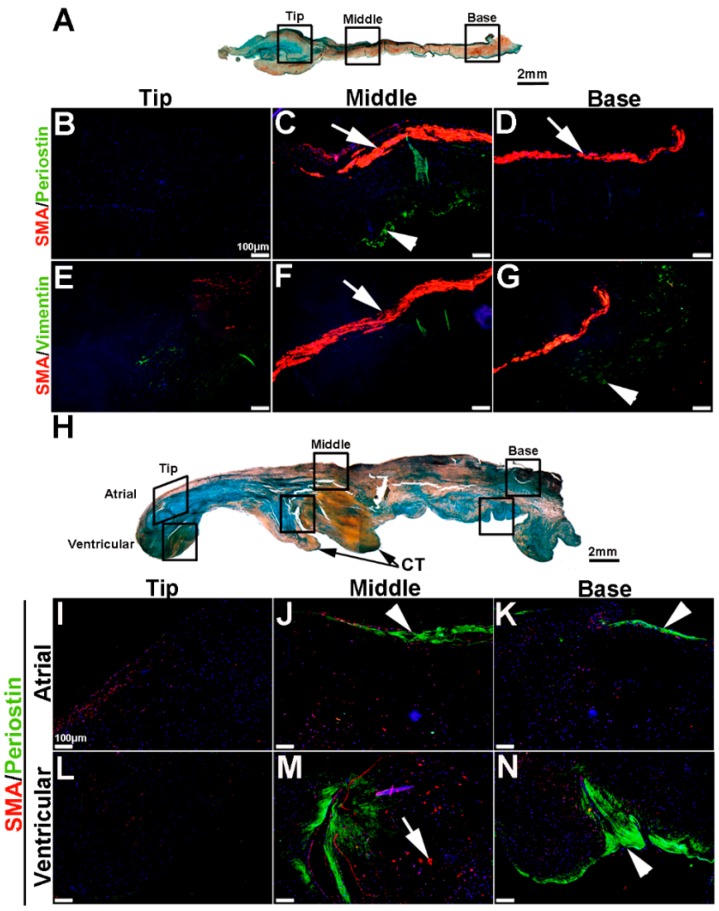

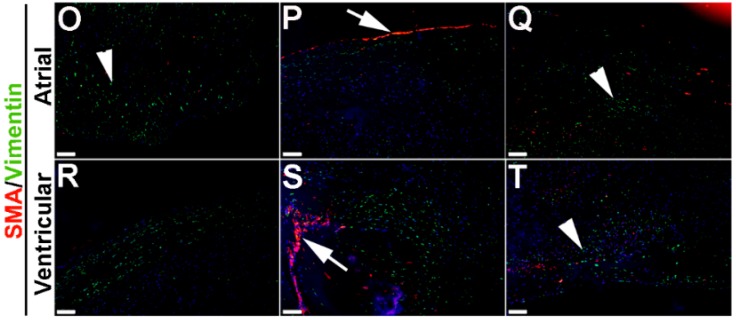
VIC phenotypes in diseased human myxomatous mitral valve biopsies. (**A**,**H**) Pentachrome staining to show extracellular matrix composition and organization in control (**A**) and myxomatous (**H**) mitral valves from human patients. (**B**–**G**,**I**–**T**) Immunohistochemistry to detect expression of smooth muscle α-actin (SMA) with Periostin (**B**–**D**,**I**–**N**) and Vimentin (**E**–**G**,**O**–**T**) in cells within the mitral valve leaflet of control (**B**–**G**) and myxomatous (**I**–**T**) patients. Arrows indicate SMA expression (red) and arrowheads indicate Periostin (green) expression. Boxed areas in A and H highlight the regions of immunostaining of cells within the tip, middle and base regions of both control and diseased valves. CT, chordae tendineae.

To extend these studies into humans, expression patterns were examined in control and myxomatous mitral valve biopsies from human patients (male, aged >50 years old). By Pentachrome staining, myxomatous valves are histologically thickened with excess abundance of proteoglycans (Figure 8H) as compared to control subjects that died from subarachnoid hemorrhage or intracranial bleeds (Figure 8A). Due to the large size, human mitral valves were divided into top, middle and base regions (See Figure 8A,H) and myxomatous valves were further subdivided into atrial and ventricular zones (Figure 8H). Control mitral valves express high levels of SMA along the atrial surface (arrowheads Figure 8C,D,F,G) with Periostin enriched on the ventricular side, particularly at the base (Figure 8D). Little staining of any marker, other than Vimentin is observed in the tip region (Figure 8E). In myxomatous valves, SMA expression on the atrial surface is diminished, although expression is noted in VICs within the middle and base regions (Figure 8J,K,M,N). Periostin is significantly increased at the atrial (Figure 8K) and ventricular (Figure 8N) surfaces in disease, and Vimentin reactivity remains throughout the valve leaflet (Figure 8O,R). These observations suggest that SMA and Periostin are differentially expressed in human myxomatous mitral valves which may reflect phenotypic changes in the VIC population.

## 4. Discussion

The phenotypic characterization of VICs is incomplete and therefore the goal of this study was to use molecular markers to “road map” the VIC population in embryonic, postnatal and adult heart valves from wild type and diseased mice. In 2007, a review by Liu *et al.*, described five phenotypes of the VIC population: embryonic progenitor endothelial/mesenchyme cells, quiescent, activated, progenitor and osteoblastic. Here, we extend these studies and demonstrate the complex, age-, species- and disease-dependent heterogeneity of VICs within the valve structures. It is apparent that eVICs are dynamic: in the endocardial cushion the newly transformed cells are considered mesenchymal in phenotypes based on their molecular profiles and morphology (reviewed in [44]). However, it should be noted that even at this stage, studies have demonstrated heterogeneity as well as differentiation beyond mesenchyme phenotype in subsets of cushion cells [45]. Interestingly, SMA is expressed in newly transformed mesenchyme cells from endocardial cushion assays *in vitro* [46,47,48,49,50,51], yet, *in vivo*, we detected only low levels of SMA expression at E12.5 (Figure 1). It can be speculated that this discrepancy could be due to the *in vitro* environment that favors SMA induction, including growth factor enriched media (containing Tgfβ) and/or artificial biomechanical stiffness. Beyond the cushion stage, the nomenclature of interstitial cells has not been well defined. Here we show that SMA is expressed in subsets of cells within the elongating superior cushion and parietal leaflets at E13.5 (Figure 1D–F) and along the atrial surface of the valve from E17.5 (Figure 2 and Figure 3) to PND5 in mice. These SMA-positive cells may represent a myofibroblast-like phenotype that promotes physiological remodeling of the valve primordia during development and tri-laminar formation after birth [1,5]. In contrast, the Perisotin-positive cells at late embryonic and postnatal stages are distinct from the myofibroblasts and may represent fibroblast- or protofibroblast-like phenotypes. There are also many cells that do not express SMA or Periostin, but are Vimentin positive, suggesting an alternative phenotype. However, the ubiquitous expression pattern of Vimentin until late postnatal stages suggest that this is a generic and less informative marker of the ever-changing VIC population. Although, Vimentin along with Twist1 have previously been noted as markers of mesenchyme cells, yet we observe additional expression in the endothelium and therefore these should proteins be considered as endothelial and interstially expressed. The nomenclature of interstitial cells within the developing valve beyond the endocardial cushion stage has not been assigned, but the dynamic heterogeneity of these cells suggests maturation beyond mesenchyme cells types and therefore eVIC deems appropriate. As described [43], adult VICs in the mouse are quiescent (qVICs) with rare SMA expression observed and undetectable Periostin (Figure 4A,F). However in adult human valves, we observed high levels of SMA expression along the atrial surface and Periostin enriched at the ventricular surface (Figure 8), suggesting species dependency. These expression profiles of postnatal VICs *in vivo* are different to cultured rMVICs (Figure 5) but somewhat similar to mesenchyme cushion cells at E12.5 (Figure 1). However, adult qVICs have lower proliferation and migratory indices and are functionally diverse from cushion mesenchyme cells and therefore further work is required to phenotypically characterize adult qVICs at molecular and functional levels.

Valve disease is commonly associated with VIC activation of otherwise quiescent cells. The gold standard marker of these cells has been SMA, however increased expression is not always observed in disease [17,38,39]. Therefore, it is considered that pathological ECM remodeling by VICs may not require SMA expression, or as our data suggests there are additional markers of disease-causing VICs *in vivo*; many of which remain unknown. Here, we show that VICs from human patients and two mouse models of mitral valve disease express diverse molecular profiles, yet commonly exhibit myxomatous phenotypes associated with excess proteoglycan accumulation. In 15 month old *Filamin A^−/−^* mice (Figure 5D) SMA is highly expressed in VICs throughout the valve leaflets, while increased SMA in affected mitral valves from *Fbn1^C1039/+^* mice is localized to atrial regions (Figure 6D and Figure 7D).

In addition, Periostin is not significantly expressed in *Filamin A^−/−^* mice (Figure 6D), but highly detected in *Fbn1^C1039/+^* mitral valves (Figure 7D), similar to human disease (Figure 8). Twist1 was not highly detectable in healthy, adult or cultured VICs, yet it is observed in a small subset of VICs in *Filamin A^−/−^* mice (Figure 6F) and in VECs and sub-endothelial VICs in the *Fbn1^C1039/+^* model (Figure 7F), characteristic of more mesenchyme-type phenotypes. These discrepancies between disease models could be due to the differential pathological stimuli underlying mitral valve disease in these mice. For example, substitute mutations in *Fbn1* and increased Tgfβ signaling are thought to promote changes in mitral valve structure in *Fbn1^C1039/+^* mice [40], while rescue of altered cross-talk between the cytoskeleton protein *Filamin A,* with decreased serotonylation of Filamin A underlies mitral valve abnormalities in deficient mice [41]. The take home message from these studies suggests that despite similar mitral valve phenotypes, the VIC molecular profiles are diverse. In contrast to this conclusion, increased SMA is common to both calcific and myxomatous disease types, yet in these states the biomechanical phenotypes of the valve are very different; stenotic *versus* prolapsed [6,8]. The reason for this is not clear, but in calcific aortic valve disease deposition of the mineralized ECM is driven by VICs expressing osteoblast markers that precede increased SMA expression *in vitro* [36,37]. A comparative post-SMA-positive VIC in myxomatous disease has not been described but warrants further investigation.

While this current descriptive study and others previous are highly informative they do not delineate the differences between cause and effect of VIC phenotypes on disease processes. Studies by Rabkin *et al.* support a causative role for SMA-positive VICs in driving pathological changes in the valve ECM [6]. However, it remains unclear what induces SMA in disease and we are left to speculate that localized changes in the hemodynamic environment as a result of structural defects, or biomechanics following pathologic ECM remodeling might play a role [37]. Furthermore it is considered that SMA may not only mark resident aVICs but also represent newly transformed mesenchymal cells recently shown to be present in disease as a result of adult EMT [33,50,52]. However, data shown here (Figure 1) suggests that SMA is not highly expressed in EMT-derived cells within the endocardial cushion and this would therefore suggest that pathological-EMT may not recapitulate developmental events, although further work is required to determine this.

## 5. Conclusions

In conclusion, these studies have highlighted the heterogeneity of VIC phenotypes in developing and maturing mitral valve structures in healthy mice over time. Furthermore, we have identified that increased SMA expression is not commonly observed in mouse models of mitral valve disease and other cell lineages (mesenchyme-, fibroblast-like) may also contribute to valve disease pathology. However, it is recognized that aortic valve disease was not included in these studies and due to the differential hemodynamic environment and structure VIC behavior may be different in the outflow valve sets. For advancements in the treatment of valve disease to occur, it is imperative that we delineate the function of the diverse VIC populations in disease types and identify the pathological stimuli promoting phenotypic changes in VICs. Furthermore, understanding the plasticity of adult VICs in disease will determine if promoting quiescence would be an effective approach in the development of non-surgical therapeutics. In addition, as age, sex and disease state have all been shown to influence VIC phenotypes, the need for personalized medicine will be essential when considering diagnostic and prognostic treatment programs.
